# Metformin protects prepubertal mice ovarian reserve against cyclophosphamide via regulation of the PI3K/Akt/mTOR signaling pathway and *Yap-1*

**DOI:** 10.1186/s13048-024-01572-4

**Published:** 2024-12-19

**Authors:** Negin Zatalian, Azam Dalman, Parvaneh Afsharian, Maryam Hezavehei, Hamid Gourabi

**Affiliations:** 1 Department of Molecular Cell Biology-Genetics, Faculty of Basic Sciences and Advanced Technologies in Biology, University of Science and Culture, Tehran, Iran; 2 Department of Embryology, Reproductive Biomedicine Research Center, Royan Institute for Reproductive Biomedicine, ACECR, No. 2, Hafez St., Banihashem St., Resalat Ave., Tehran, 16635-148 Iran; 3 Department of Genetics, Reproductive Biomedicine Research Center, Royan Institute for Reproductive Biomedicine, ACECR, No. 2, Hafez St., Banihashem St., Resalat Ave., Tehran, 16635-148 Iran; 4 Research Center for Reproduction and Fertility, Faculty of Veterinary Medicine, Montreal University, St-Hyacinthe, QC Canada

**Keywords:** Metformin, Cyclophosphamide, Ovarian reserve, PI3K/Akt/mTOR pathway

## Abstract

**Background:**

Cyclophosphamide is a widely utilized chemotherapeutic agent for pediatric cancers, known to elicit adverse effects, including perturbation of the PI3K/Akt/mTOR and Hippo signaling pathways, thereby diminishing ovarian reserve and fertility potential in females. Consequently, this investigation delves into the mitigative effects of metformin on cyclophosphamide-induced ovarian impairment in prepubertal mice.

**Methods:**

Twenty-four 14-day-old NMRI female mice were distributed into four groups: Control (Cont), Cyclophosphamide (Cyc), Metformin (Met), and Metformin plus Cyclophosphamide (Met-Cyc). The Met-Cyc group was given daily doses of 150 mg/kg metformin for 11 consecutive days and in parallel 3 intermittent doses of 65 mg/kg cyclophosphamide once every three days. The Met and Cyc groups were given identical doses of Met or Cyc alone. The control group received normal saline treatment. On the 12^th^ day, mice were sacrificed for analysis. Stereological methods were employed to measure the overall volume of the ovaries, including the medulla, cortex, and follicles, along with measuring anti-Müllerian hormone (AMH) levels using an ELISA kit. Furthermore, qRT-PCR was utilized to quantify the expression levels of genes, including *P53*, *Bax*, *Bcl-2*, *Rad-51*, *Pten*, *Mtor*, and *Yap-1*.

**Results:**

The findings demonstrate that metformin ameliorates cyclophosphamide-induced ovarian toxicity by increasing AMH levels and attenuating the excessive activation of primordial follicles, the ratio of growing to quiescent follicles, and follicular atresia. This protective effect is mediated by the downregulation of apoptosis-related genes, upregulation of the gene involved in a reparative pathway, and modulation of the PI3K/Akt/mTOR pathway evidenced by increased expression of *Pten*, *Mtor* and Hippo pathway by *Yap-1* expression.

**Conclusions:**

Our results advocate for the potential of metformin as a viable therapeutic option for preserving ovarian function in cyclophosphamide-treated adolescent girls, given its favorable side effect profile and ability to improve cyclophosphamide-induced ovarian damage.

## Background

Despite advancements in cancer diagnosis and treatment leading to increased survival rates, the reproductive system can be adversely affected by treatment-related side effects. Chemotherapy, often pivotal to cancer treatment, carries the risk of infertility, particularly in female survivors, attributed to premature ovarian insufficiency (POI) [1]. Among chemotherapy agents, cyclophosphamide is commonly employed in the treatment of childhood cancers, myeloma, breast cancer, and autoimmune disorders [2]. Cyclophosphamide diminishes ovarian reserve through multiple mechanisms. It triggers hyper-activation of primordial follicles by instigating phosphorylation of the PI3K/Akt/mTOR (Phosphoinositide-3-kinase/ Protein kinase B/ mammalian target of rapamycin) and Hippo signaling pathways [3, 4] or induces apoptosis in growing follicles, leading to a reduction in anti-Müllerian hormone (AMH) levels, which serves as a suppressor of primordial follicle activation and is secreted by granulosa cells of growing follicles [5]. Luan et al. elucidated cyclophosphamide’s activation of apoptosis pathways in primordial germ cells within the ovarian reserve [6]. Furthermore, robust experimental and clinical evidence underscores that alkylating agents such as cyclophosphamide, which disrupt DNA integrity, can engender severe damage to reproductive organs, including stromal and microvascular impairments [7].

Ovarian tissue cryopreservation stands as the sole viable option for fertility preservation among pubertal girls undergoing gonadotoxic chemotherapy [1]. The clinical approach to restoring fertility through cryopreserved ovarian tissue involves transplanting the tissue back into the patient [8]. However, concerns arise regarding the risk of reintroducing malignant cells into the transplanted tissue, potentially stemming from the transfer of metastatic cells into the ovarian tissue. Additionally, it is crucial to take into account age restrictions for this procedure [9, 10]. Notably, another important limitation involves the loss of follicles [11]. Hence, the utilization of compounds possessing antioxidant and cytoprotective properties to shield ovarian function from chemotherapy-induced harm emerges as a pivotal advantage [12]. A pioneering study by Castrillon et al. elucidated that FOXO3a^−/−^ (Forkhead Box O3) prevents primordial follicle recruitment [13]. Subsequently, Kalich-Philosoph et al. demonstrated in 2013, through investigations into the phosphorylation of the PI3K/Akt/mTOR pathway, that AS101 can mitigate the reduction of ovarian reserve in cyclophosphamide-treated mice [14]. Furthermore, the specific mTORC1 inhibitor rapamycin has been shown to sustain follicular reserve by thwarting the hyper-activation of primordial follicles via the PI3K/Akt/mTOR signaling pathway and diminishing apoptosis in growing follicles [3]. Feng et al. demonstrated that melatonin treatment substantially curbed cyclophosphamide-induced hyper-activation of primordial follicles by preserving plasma AMH levels, consequently averting a decrease in litter size in cyclophosphamide-treated mice. Moreover, melatonin exhibited protective effects against ovarian granulosa cell loss by suppressing the mitochondrial apoptotic pathway [5].

Many cancer cells respond relatively well when first exposed to chemotherapy drugs; however, some patients eventually develop resistance to these agents. Consequently, acquired resistance to chemotherapy poses a significant hurdle to effective cancer treatment [15]. Evidence suggests that the PI3K/Akt/mTOR and Hippo pathways are pivotal in conferring resistance of cancer cells to chemotherapy; hence, modulation of these pathways could represent a promising approach for cancer therapy [15, 16]. It appears that, in order to preserve ovarian reserve, it is necessary to use substances that do not interfere with cancer treatment.

Metformin, a widely used drug for managing type 2 diabetes, exhibits the ability to impede the growth and metastasis of various tumor cell types [17]. Mechanistically, metformin triggers the activation of AMP-activated protein kinase (AMPK) by inhibiting the electron transport chain, resulting in decreased ATP levels and elevated AMP levels, thereby stimulating AMPK activation [17]. Once activated, AMPK exerts inhibitory effects on mTOR and YAP/TAZ (Yes-Associated Protein/ transcriptional coactivator with PDZ-binding) signaling pathways [18]. Studies have revealed that metformin administration in cancer cells suppresses growth and proliferation while promoting apoptosis [19]. In a recent study by Huang et al. in 2021, it was demonstrated that metformin preserves the ovarian reserve in adult mice treated with cyclophosphamide through the mTOR inhibitory pathway, consequently enhancing cell proliferation and triggering an AMPK/p53/p21-mediated apoptotic response [20]. Moreover, in 2024, Yang et al. illustrated that metformin treatment in adult premature ovarian failure (POF) mice improves follicle count and hormonal balance while reducing oxidative stress. Additionally, metformin was found to mitigate inflammatory responses and reduce reactive oxygen species (ROS) accumulation in primary granulosa cells through the AMPK/PPAR-γ/SIRT1 pathway [21].

Gonadal protection in prepubertal females treated with cyclophosphamide is essential. In this study, we investigated the protective efficacy of metformin against the hyper-activation of primordial follicles induced by cyclophosphamide through PI3K/Akt/mTOR signaling pathway and *Yap-1* from Hippo pathway, utilizing a chemotherapy mouse model.

## Methods

### Mice

Fourteen-day-old female NMRI mice were obtained from the Royan Institute laboratory animal breeding center (Tehran, Iran) and housed under controlled conditions, including a constant temperature of 20–26 °C, relative humidity of 30–70%, a 12-h light-dark cycle, and free access to sterile food and water. The Ethics Committee of the Royan Institute approved the experimental protocols.

### Experimental design

**Fig. 1:**
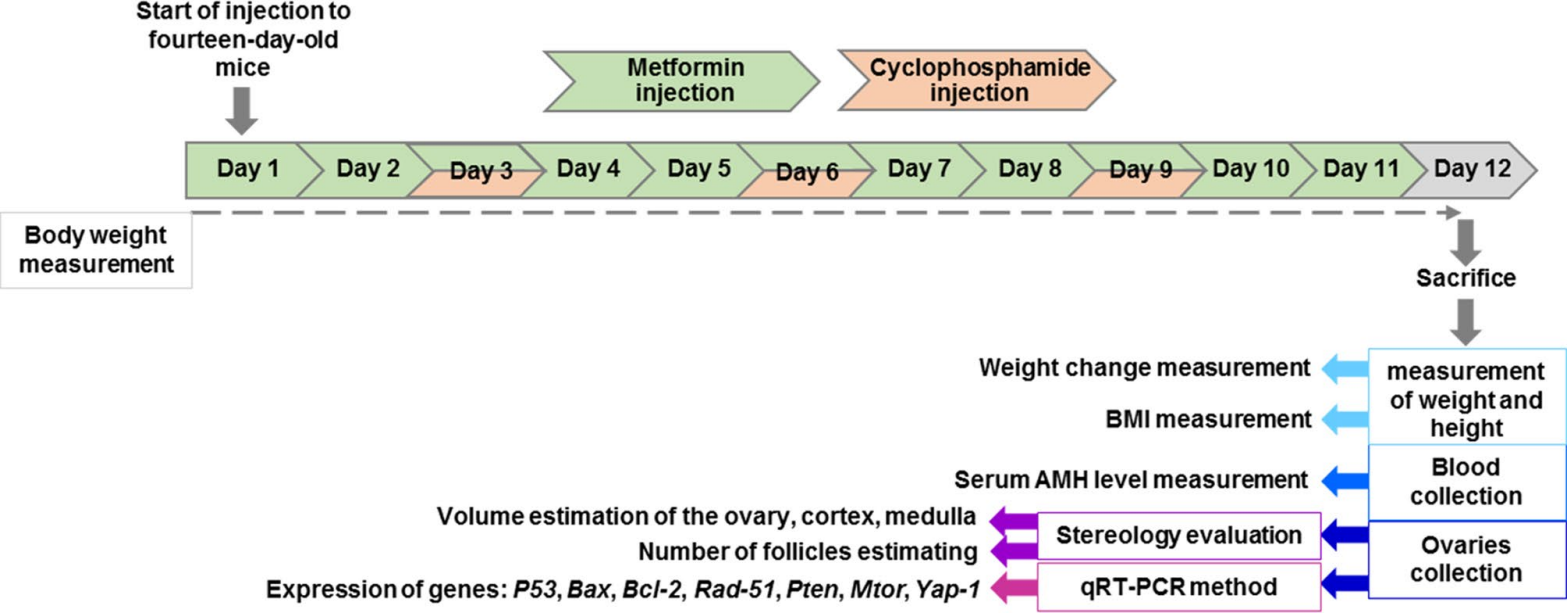
Mice treatment timeline. Experimental design diagram

Cyclophosphamide (Chandra Bhagat Pharma, India) and metformin-hydrochloride (Sigma Aldrich, USA) were dissolved in normal saline immediately before injection. The experimental design consisted of 24 mice that were divided into four groups (*n* = 6 per group): the control group (Cont) received 100 µl of normal saline for 11 consecutive days, the metformin group (Met) received an IP injection of 150 mg/kg body weight for 11 consecutive days, the cyclophosphamide group (Cyc) received an IP injection of 65 mg/kg body weight on the third, sixth, and ninth days, and the metformin plus cyclophosphamide group (Met-Cyc) received a dose of 150 mg/kg body weight of metformin for 11 consecutive days, initiated two days before the start of cyclophosphamide injection, and a dose of 65 mg/kg body weight of cyclophosphamide on the third, sixth, and ninth days (Fig. 1). Metformin dosage was calculated using a human equivalent dose formula based on 500 mg/kg typical human metformin dose [22]. The dose of cyclophosphamide and duration of metformin and cyclophosphamide were determined via administration of different doses and intervals and paying attention to the histological results.

During the experiment, all mice were weighed daily before injection. On the 12^th^ day, the mice were anesthetized by IP injection of 10% ketamine and 2% xylazine. Blood samples were immediately collected from the mice’s hearts to analyze biochemical factors such as AMH. Following blood collection, the mice were sacrificed, and their body lengths were measured to calculate body mass index (BMI).

After blood coagulation, blood samples were centrifuged (Sigma 2–16 KL Refrigerated centrifuge, USA) at 3000 rpm for 5 min to separate the serum. Sera were then stored at -70 °C. In addition, the ovaries were collected; the right ovary of each mouse was placed in Bouin’s fixative to fix the tissue for stereology evaluation, and the left ovary was placed in RNA Later (ThermoFisher, USA) and stored at -70 °C to examine gene expression by qRT-PCR method.

### BMI and weight change measurement

Weight changes were determined by comparing the mice’s weights on the final day with those on the initial day. Additionally, The height of mice was measured to calculate BMI, and BMI was calculated using Eq. 1 [23].

1 $$\:BMI\:=\frac{body\hspace{0.33em}weight\hspace{0.33em}\left(kg\right)}{{\left(nose\hspace{0.33em}to\hspace{0.33em}anus\hspace{0.33em}lenght\hspace{0.33em}\left(m\right)\right)}^{2}}$$

### Serum AMH level measurement

AMH levels were assessed in all four groups. Serum AMH concentration was measured using a mouse AMH ELISA kit (Abbexa, UK) per the manufacturer’s instructions.

### Tissue preparation and stereological study

For the stereological study, the right ovary was excised, weighed, and immersed in Bouin’s solution for 2 h. Subsequently, it was transferred to 10% formalin solution and kept at 4 °C for 2 days. Following tissue processing, the samples were embedded in paraffin blocks for further analysis.

The orientation method was employed to acquire isotropic uniform random (IUR) sections [24]. Subsequently, consecutive sections measuring 5 and 20 μm in thickness were cut using a microtome. These sections were then stained with hematoxylin and eosin (H&E) (Bio Optica, Italy) [24, 25].

### Volume estimation of the ovary, cortex, and medulla

The total volume of the ovary was estimated utilizing the Cavalieri method [24]. In brief, 5 μm thick sections were selected using systematic random sampling and examined under a microscope at a magnification of ×10 (BX51, Olympus, Japan). A counting probe was randomly superimposed on the images, and points were counted accordingly. Subsequently, the total volume of the ovary was estimated through Eq. 2 [24, 25].

2 $$\:Vtotal\hspace{0.33em}ovary={\sum\:}_{i=1}^{n}P\times\:a\left(p\right)\times\:t$$

where, $\:{\sum\:}_{i=1}^{n}P$ denotes the total number of points counted in 12 sections, a(p) represents the area associated with each point, and (t) is the distance between the sections.

The volume density of the ovary compartments was calculated employing the point counting method and Eq. 3:

3 $$\:Vvcortex/medulla\:=\frac{{\sum\:}_{i=1}^{n}Pmedulla/cortex}{{\sum\:}_{i=1}^{n}Ptotal}$$

where $\:{\sum\:}_{i=1}^{n}Pmedulla/⥂cortex$donates the number of counted points hitting the medulla/cortex and $\:{\sum\:}_{i=1}^{n}Ptotal$ is the total number of counted points hitting the ovary sections.

Finally, the volume of the medulla and cortex was determined by multiplying the volume density (Vv) by the total volume of the ovary as per Eq. 4:

4 $$\:Vmedulla/cortex\:=Vtotal\times\:Vvcortex/medulla$$

### Number of follicles estimating

The optical dissector method was employed to estimate the number of follicles [24]. Specifically, 20 μm thick sections were selected via systematic random sampling. These sections were examined using an Olympus microscope (model BX41TE) with 100x magnification, equipped with a microcator (ND 221 B, Heidenhain, Germany) connected to a computer and a probe. Nuclei of follicular cells were sampled utilizing an unbiased counting frame, ensuring no contact with the forbidden lines of the probe. The number density (Nv) of different types of follicles was calculated using Eq. 5.

5 $$\:NV=\frac{{\sum\:}_{i=1}^{n}Q}{a/f\times\:h\times\:{\sum\:}_{i=1}^{n}P}$$

where $\:{\sum\:}_{i=1}^{n}Q$ donates the total number of counted follicles, ($\:a/f$) is the area of ​​the counting frame, $\:h$ represents the tissue thickness considered for counting, and $\:{\sum\:}_{i=1}^{n}P$ is the total number of points superimposed on the selected fields. Finally, the result of the equation is then multiplied by the total volume ovary to obtain the total number of follicles.

Follicles were categorized into four distinct types: primordial follicles, characterized by a flat layer of pre-granulosa cells surrounding the oocyte; primary follicles, encompassing the oocyte enveloped by a cuboidal layer of granulosa cells; secondary follicles, defined by the presence of at least two layers of granulosa cells without an antral cavity; and antral follicles, distinguished by several layers of granulosa cells surrounding the oocyte along with the presence of an antral cavity. Any follicles exhibiting abnormalities or containing pyknotic granulosa cells were classified as atretic follicles [5].

### RNA extraction and quantitative real-time PCR (qRT-PCR)

Total RNA was extracted from each ovary using Trizol (QIAGEN, Germany), and genomic DNA was removed using a Genomic DNA Removal Kit (Thermo Fisher Scientific, USA). RNA quality and purity were evaluated using a NanoDrop spectrophotometer (Thermo Fisher Scientific, USA) with an absorbance of 260/280 nm and agarose gel electrophoresis (Bruker BioSpin, USA). RNA was reverse transcribed into cDNA through a cDNA synthesis kit (SMOBio, USA). RT-qPCR was subsequently performed in an ABI StepOnePlus real-time PCR system (Applied Biosystem, USA) using the following protocol: 2 µL of diluted cDNA, 2.5 µL of SYBR ​​Green Master Mix (Amplicon, UK), 3.5 µL dH2O, 1 µL of forward primer and 1 µL of reverse primer. Primers were obtained from the primer bank of the Royan Institute. Primers include *Pten*, *Mtor*, *Yap-1*, *Rad-51*, *P53*, *Bax*, *Bcl-2*, and *Gapdh*. The sequences of the primers are listed in Table 1. The PCR cycle was performed according to the following temperature schedule: holding stage (95 °C for 10 min), cycling stage followed by 40 PCR cycles (95 °C for 15 s and 60 °C for 60 s) and Melt Curve stage (95 °C for 15 s, 60 °C for 60 s and 95 °C for 15 s). Subsequent to gene expression normalization to the housekeeping gene (*Gapdh*), fold changes were calculated relative to the control group using a 2^−∆∆Ct^ method.

**Table 1:** List of forward and reverse primer sequence

Gene	Forward primer sequence	Reverse primer sequence	Product size (bp)
*Pten*	AGAGACATTATGACACCGCC	ATTACACCAGTCCGTCCCT	184
*Mtor*	CGCCTTCACAGATACCCAG	TAGACCTTAAACTCCGACCTC	139
*Yap1*	CAATGACAACCAATAGTTCCGA	TTTCATCCACACTGTTGAGG	141
*Rad51*	TCAACACAGACCACCAGAC	CGACACCAAACTCATCAGCA	201
*P53*	AACTTACCAGGGCAACTATG	TGTGCTGTGACTTCTTGTAG	203
*Bax*	TTGCTACAGGGTTTCATCCAG	CCAGTTGAAGTTGCCATCAG	246
*Bcl-2*	GCCTTCTTTGAGTTCGGT	ATATAGTTCCACAAAGGCATCC	161
*Gapdh*	GACTTCAACAGCAACTCCCAC	TCCACCACCCTGTTGCTGTA	125

### Statistical analysis

Statistical analysis was conducted using GraphPad Prism software (version 8, GraphPad Software, San Diego, CA). A one-way ANOVA was employed to compare results across different groups, with differences between the two groups analyzed using Tukey’s multiple comparison test. Descriptive statistics were presented as mean ± standard deviation (SD), and *p*-values less than 0.05 (*P* < 0.05) were considered statistically significant.

## Results

### Cyclophosphamide-induced reduction in weight gain

**Fig. 2:**
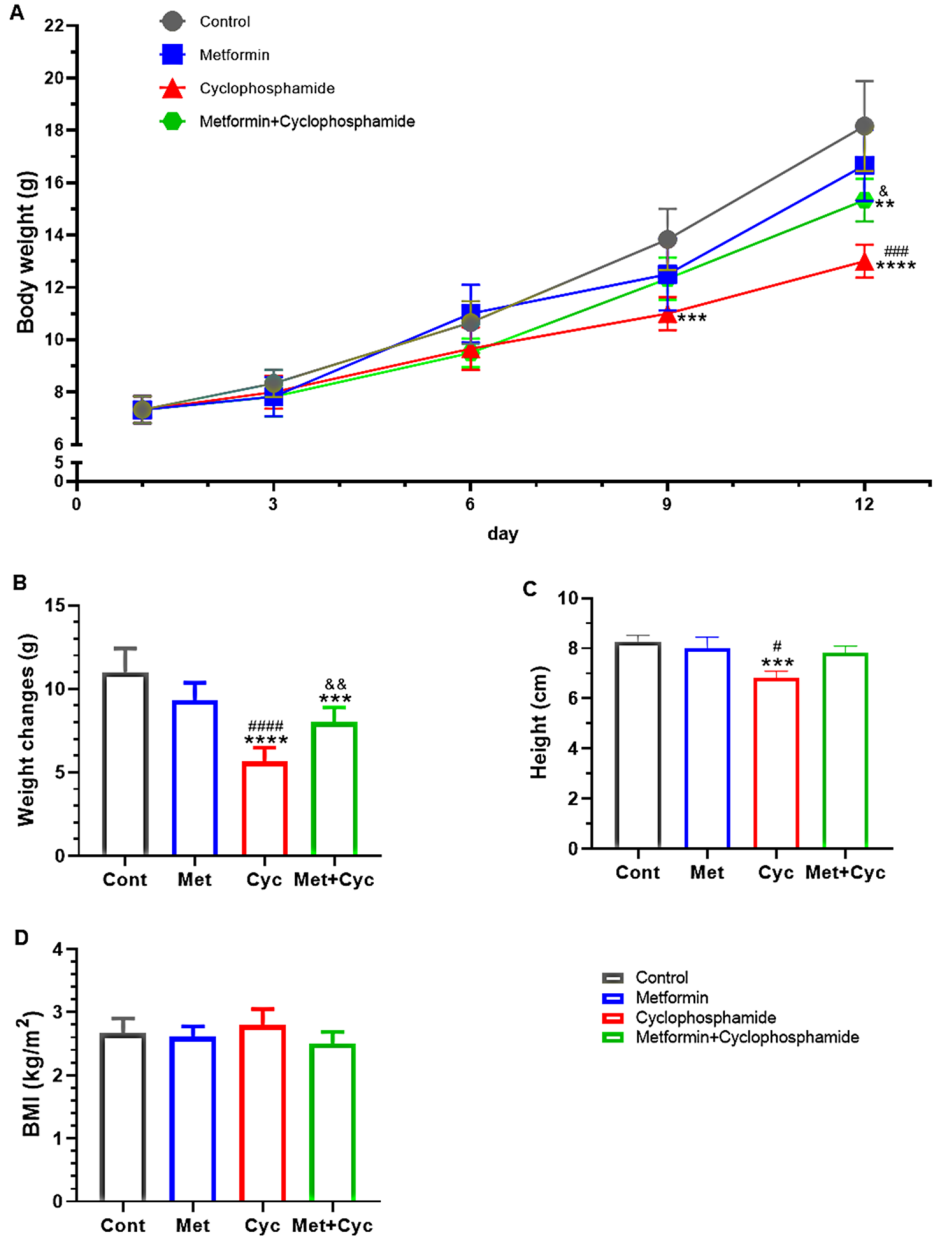
Body weight gain, weight changes, height and BMI. (**A**) Weight gain trend in four groups. (**B**) Weight changes in different groups: the weight difference was calculated on the first and last days. (**C**). Height difference in four treatment groups. (**D**). BMI information of the experimental groups. Data are expressed as the mean ± SD. ^**^P < 0.01, ^***^P < 0.001 and ^****^P < 0.0001, compared with the Cont group; ^#^*P<0.05,* ^###^*P* < 0.001 and ^####^*P* < 0.0001, compared with the Met group, ^&^*p* < 0.05 and ^&&^*p* < 0.01, compared with the Cyc group

The weight differences of mice on the 1^st^, 3^rd^, 6^th^, 9^th^, and 12^th^ days were compared, as shown in Fig. 2A. On the 9^th^ day, the Cyc group mice weighted significantly less than the Cont group mice (11.000 ± 0.6325 versus 13.830 ± 1.16 g, *P* = 0.0007). Furthermore, on the final day (12th) of cyclophosphamide treatment, a significant weight reduction was observed in comparison to the Cont, Met, and Met + Cyc groups (13.000 ± 0.6325 versus 18.170 ± 1.722, 16.670 ± 1.366, and 15.330 ± 0.8165 g, respectively; *P* < 0.0001, *P* = 0.0002, and *P* = 0.0163). Additionally, the Met + Cyc group demonstrated a significant difference compared to the Cont group (*P* = 0.0033, Fig. 2A).

Comparison of the weight of mice on the last and first day of the experiment revealed a significantly lower weight gain in the Cyc group compared to the Cont, Met, and Met + Cyc groups (5.667 ± 0.8165 versus 11.000 ± 1.414, 9.333 ± 1.033, and 8.000 ± 0.8944 g, respectively; *P* < 0.0001, *P* < 0.0001, and *P* = 0.0057). Additionally, a significant decrease was observed in the Met + Cyc group compared to the Cont group (*P* = 0.0005) (Fig. 2B).

The average height of mice in the Cont and Met groups was significantly higher than in the Cyc group (8.250 ± 0.2739, 8.000 ± 0.4472 and 6.833 ± 0.2582 cm, respectively; *P* = 0.0008 and *P* = 0.0183). The Met + Cyc group averaged 7.833 ± 0.2582 cm in height, taller than Cyc group’s average but not significantly so (*P* = 0.1065) (Fig. 2C).

The comparison of BMI among the groups is depicted in Fig. 2D. No significant difference in BMI was observed among the Cont, Met, Cyc, and Met + Cyc groups (2.669 ± 0.2277, 2.608 ± 0.167, 2.794 ± 0.2541, and 2.503 ± 0.1830 kg/m2, respectively, Fig. 2D).

### Prevention of cyclophosphamide-induced reduction in blood AMH levels with metformin treatment

**Fig. 3:**
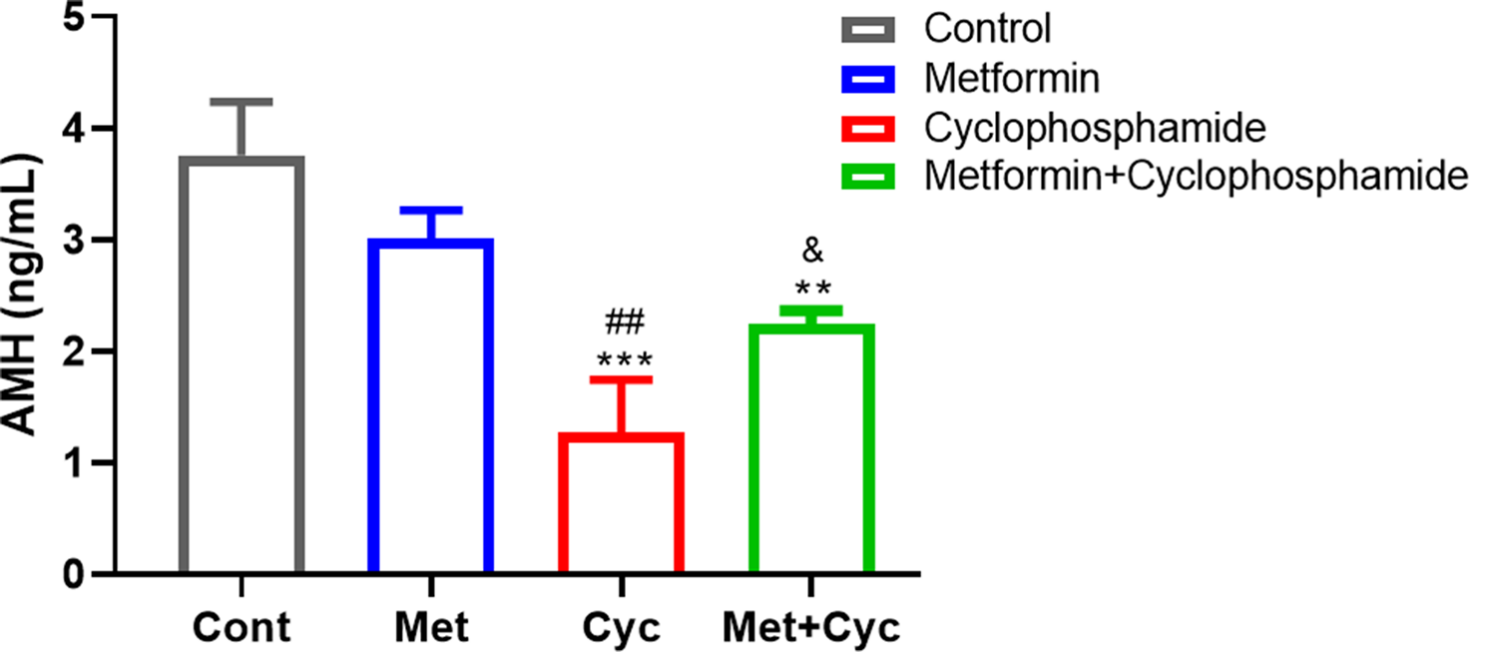
Evaluation of AMH level in the four experimental groups. Data are expressed as the mean ± SD. **P < 0.01 and ***P < 0.001, compared with the Cont group; ^##^*P* < 0.01, compared with the Met group, ^&^*p* < 0.05, compared with the Cyc group

The level of AMH was assessed, revealing the lowest levels in the Cyc group compared to the Cont, Met, and Met + Cyc groups (1.277 ± 0.4670 versus 3.757 ± 0.4788, 3.013 ± 0.2517, and 2.243 ± 0.1210 ng/ml, respectively; *P* = 0.0001, *P* = 0.0017, and *P* = 0.0457). Additionally, a significant decrease was observed in the Met + Cyc group compared to the Cont group, while no difference was noted in the Met and Met + Cyc groups (*P* = 0.0040 and *P* = 0.1165, respectively, Fig. 3).

### Improved ovarian volume in cyclophosphamide-treated mice with metformin treatment

**Fig. 4:**
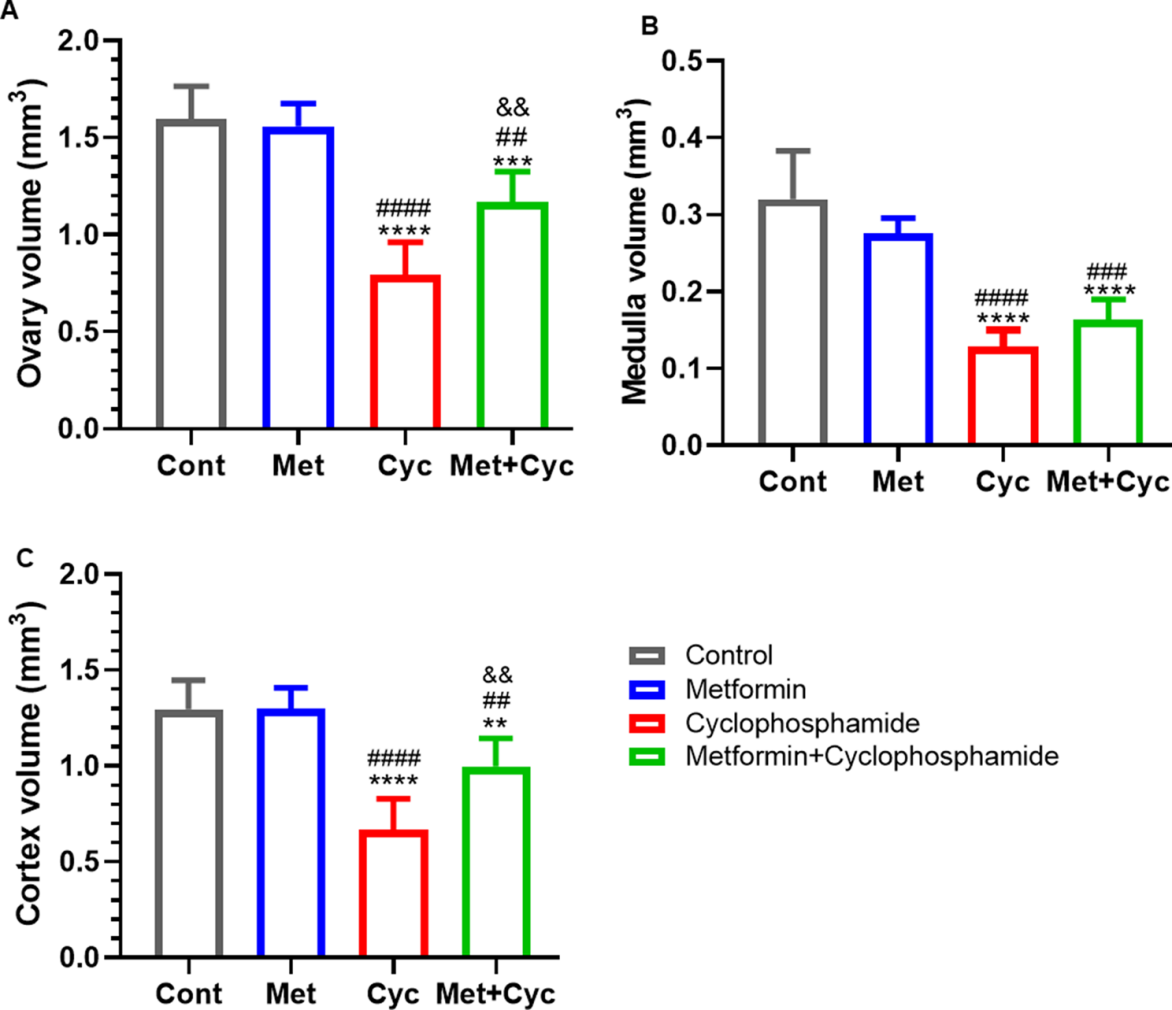
Evaluation of the total volume of the ovary, medulla, and cortex in different groups of mice. (**A**) The ovarian volume in four experimental groups. (**B**) The volume of the medulla in experimental groups. (**C**) The volume of the cortex is in four groups. Data are expressed as the mean ± SD. ^**^P < 0.01, ^***^P < 0.001 and ^****^P < 0.0001, compared with the Cont group; ^##^*P* < 0.01, ^###^*P* < 0.001 and ^####^*P* < 0.0001, compared with the Met group and ^&&^*p* < 0.01, compared with the Cyc group

According to Fig. 4A, our finding demonstrate that cyclophosphamide led to a reduction in ovarian volume compared to the Cont, Met, and Met + Cyc groups (0.7922 ± 0.1682 versus 1.596 ± 0.1679, 1.558 ± 0.1179, and 1.167 ± 0.1570 mm^3^, respectively; *P* < 0.0001, *P* < 0.0001, and *P* = 0.0037). Moreover, ovarian volume was significantly diminished in the Met + Cyc group compared to the Cont and Met groups (*P* = 0.0006 and *P* = 0.0015, Fig. 4A).

The volume of the medulla in the Cyc group was smaller compared to that in the Cont and Met groups (0.1284 ± 0.0266 versus 0.3200 ± 0.0632 and 0.2757 ± 0.0197 mm^3^, respectively; *P* < 0.0001); however, it did not differ significantly from the Met + Cyc group (0.1631 ± 0.0266 mm^3^, *P* = 0.3953, Fig. 4B).

As depicted in Fig. 4D, the volume of the cortex in mice treated with cyclophosphamide and Met + Cyc (0.6695 ± 0.1595 and 0.9982 ± 0.1455 mm^3^) was significantly smaller compared to that in the Cont, Met, and Met + Cyc groups (1.296 ± 0.1512 and 1.2999 ± 0.1092 mm^3^). Additionally, the volume of the cortex in the Met + Cyc group was significantly larger than that in the Cyc group (*P* = 0.0037, Fig. 4C).

### Preservation of ovarian reserve in cyclophosphamide-treated mice aided by metformin treatment

**Fig. 5:**
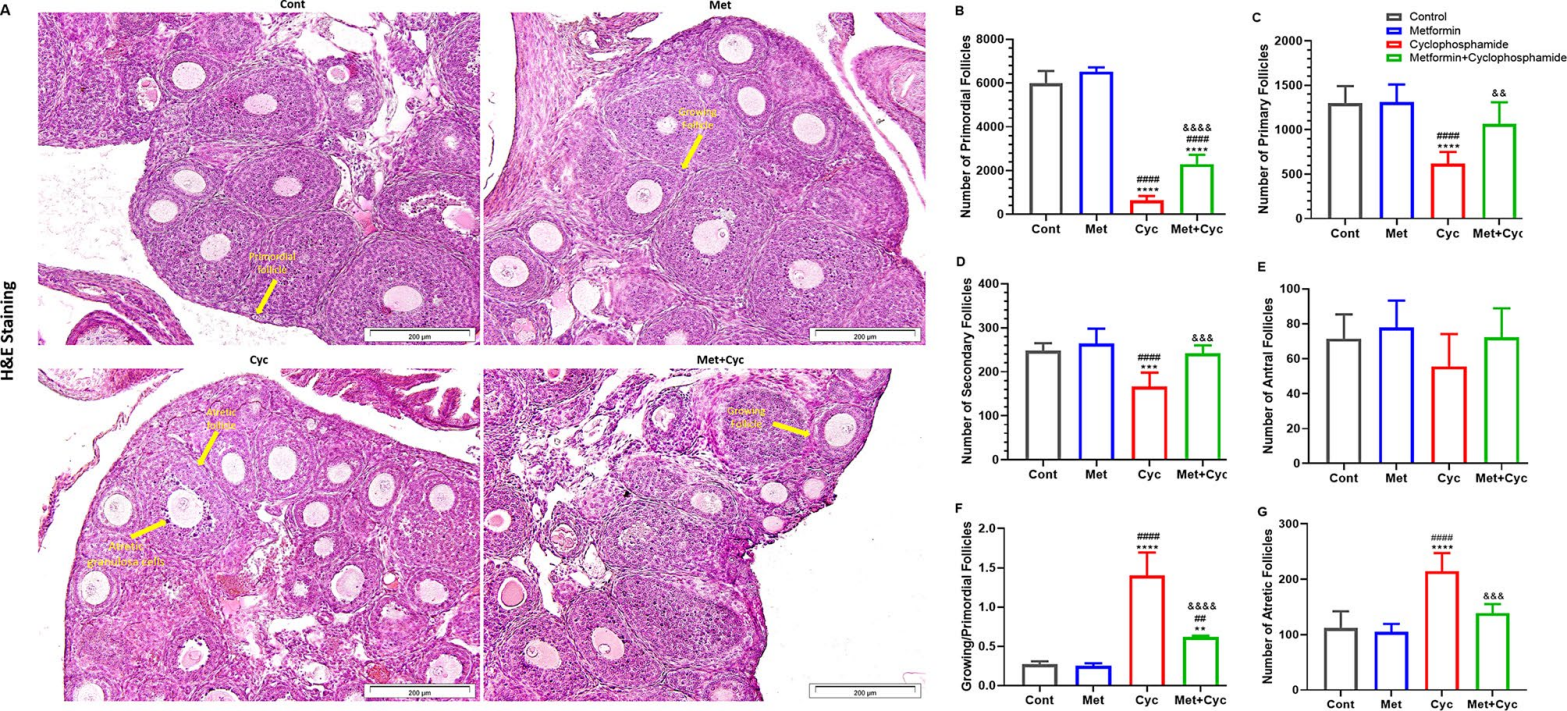
Histological section of the mice ovarian tissue in the experimental groups and follicular counts in different stages. (**A**). Staining of tissue with a thickness of 5 μm in four groups with the H&E method (the magnification is ×200 and scale bars represent 200 μm). (**B**). Comparison of the number of primordial follicles per unit volume. (**C**) Number of primary follicles per unit volume. (**D**) Number of secondary follicles per unit volume. (**E**) Number of antral follicles per unit volume. (**F**) The ratio of developing follicles to primordial follicles. (**G**) The number of atretic follicles per unit volume among 4 groups. Data are expressed as the mean ± SD. ^**^P < 0.01 and ^****^P < 0.0001, compared with the Cont group; ^##^*P* < 0.01 and ^####^*P* < 0.0001, compared with the Met group and ^&&^*p* < 0.01, ^&&&^*p* < 0.001 and ^&&&&^*p* < 0.0001 compared with the Cyc group

The ovarian tissues of mice in the four groups are demonstrated in Fig. 5 (Fig. 5A). Generally, no significant difference was observed in the number of follicles counted at different stages of folliculogenesis between the Met and Cont groups (*P* > 0.05). Primordial follicle counting revealed that cyclophosphamide led to a significant reduction in these follicles compared to the Cont and Met groups (631.1 ± 209.1 versus 5980 ± 573.0 and 6521 ± 191.3, respectively; *P* < 0.0001), while treatment with metformin in the Met + Cyc group prevented primordial follicle activation (2295 ± 425.9, *P* < 0.0001). However, there was also a statistically significant difference in the Met + Cyc group compared to the Cont and Met groups (*P* < 0.0001, Fig. 5B). The number of primary follicles in the Cyc group was significantly lower than in the Cont, Met, and Met + Cyc groups (616.9 ± 133.9 versus 1299.0 ± 192.7, 1312.0 ± 196.5, and 1064.0 ± 244.6, respectively; *P* < 0.0001, *P* < 0.0001, and *P* = 0.0040, Fig. 5C), and the number of pre-antral follicles was significantly lower in this group compared to the other three groups, Cont, Met, and Met + Cyc (166.8 ± 31.22 versus 247.9 ± 17.22, 264.6 ± 33.46, and 242.3 ± 17.88, respectively; *P* = 0.0002, *P* < 0.0001, and *P* = 0.0004, Fig. 5D). Regarding antral follicles, no significant difference was observed between groups (*P* > 0.05, Fig. 5E).

The ratio of growing follicles (primary, secondary, and antral follicles) to primordial follicles exhibited a statistically significant increase in the Cyc group compared to the Cont, Met, and Met + Cyc groups (*P* < 0.0001). Additionally, a similar increasing trend was observed in the Met + Cyc group compared to the Cont and Met groups (*P* = 0.0061 and *P* = 0.0039, respectively, Fig. 5F).

### Mitigation of cyclophosphamide-induced follicle apoptosis and improvement of reparative pathways via metformin

**Fig. 6:**
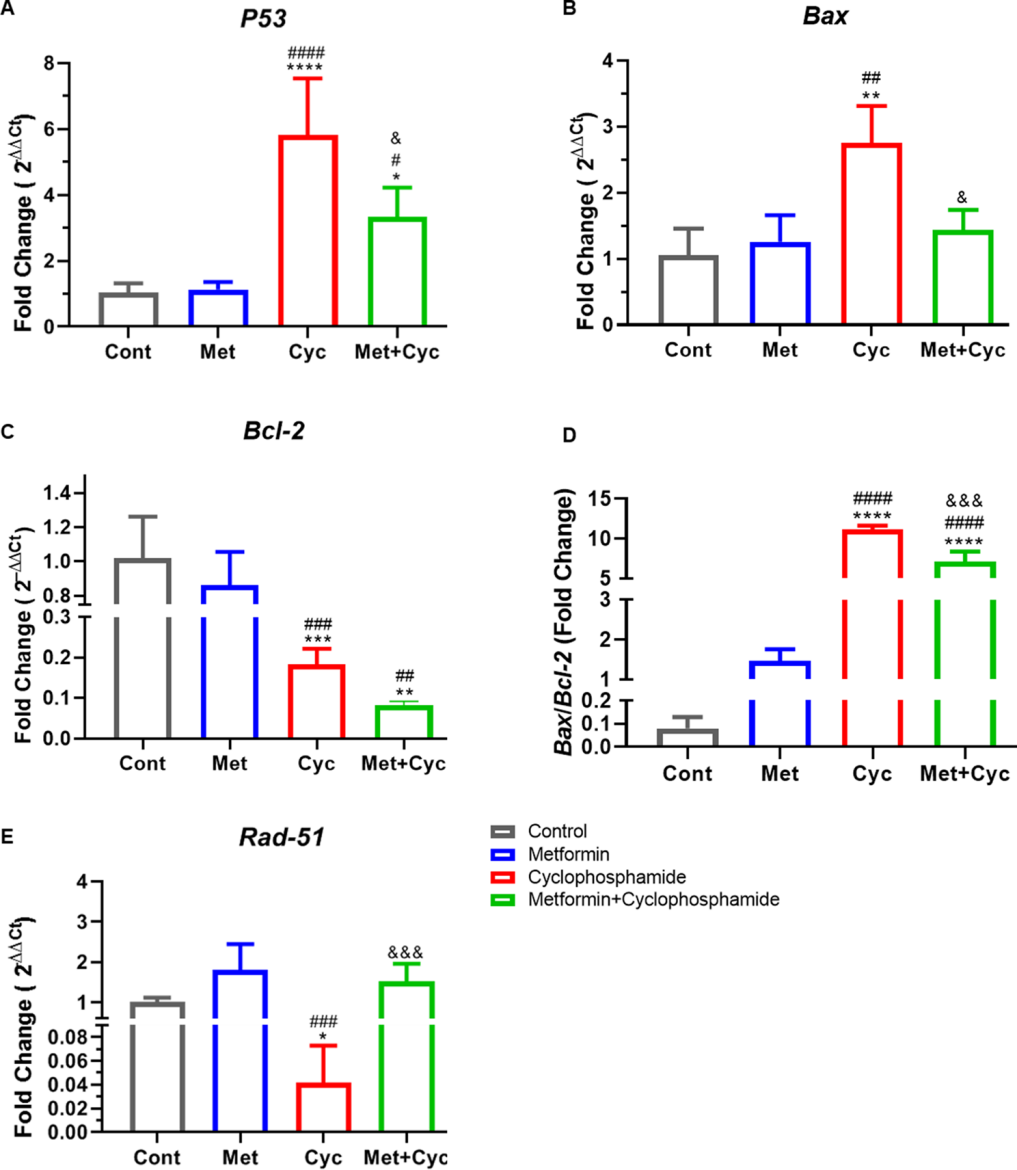
Apoptotic and repair gene expression in the four experimental groups. (**A**)*P53* gene expression, (**B**)*Bax* gene expression, (**C**)*Bcl-2* gene expression, (**D**)*Bax* to *Bcl-2* gene expression ratio and (**E**)*Rad-51*. Data are expressed as the mean ± SD. ^*^P < 0.05, ^**^P < 0.01, ^***^P < 0.001 and ^****^P < 0.0001, compared with the Cont group; ^#^*P* < 0.05, ^##^*P* < 0.01, ^###^*P* < 0.001 and ^####^*P* < 0.0001, compared with the Met group and ^&^*p* < 0.05 and ^&&&^*p* < 0.001 compared with the Cyc group

As illustrated in Fig. 5G, the highest number of atretic follicles was observed in the Cyc group, exhibiting a significant difference compared to all three groups, Cont, Met, and Met + Cyc (214.7 ± 32.58 versus 111.8 ± 30.25, 104.9 ± 14.55, and 138.7 ± 16.42, respectively; *P* < 0.0001, *P* < 0.0001, and *P* = 0.002). However, there was no significant difference among the other three groups (*P* > 0.05, Fig. 5G). Furthermore, the expression of the pro-apoptotic genes *P53* and *Bax* as well as the anti-apoptotic gene *Bcl-2*, was investigated (Fig. 6A-C). The evaluation revealed that the expression of the *P53* gene in the Cyc group was significantly increased compared to that in the Cont and Met groups (*P* < 0.0001). Treatment with metformin in combination with cyclophosphamide led to a significant decrease in the expression of this gene compared with the Cyc group (*P* = 0.0193); however, an increase in *P53* gene expression was observed in this group compared to the Cont and Met groups (*P* = 0.0198, Fig. 6A). Metformin treatment alongside cyclophosphamide significantly reduced *Bax* expression compared to the Cyc group (*P* = 0.0121, Fig. 6B). Additionally, *Bcl-2* gene expression did not significantly differ between the Cyc and Met + Cyc groups (*P* = 0.8781), but both showed a significant decrease compared to the Cont and Met groups (*P* < 0.05, Fig. 6C). Moreover, the ratio of *Bax* to *Bcl-2* gene expression indicated that the use of metformin along with cyclophosphamide decreased this ratio compared to the Cyc group (*P* = 0.0001). In contrast, this ratio significantly increased in both the Met + Cyc and Cyc groups compared to the Cont and Met groups (*P* < 0.0001, Fig. 6D). Moreover, the expression of the *Rad-51* gene, a key gene in the repair pathway, demonstrated that treatment with metformin alongside cyclophosphamide regulated the expression of this gene compared to the Cyc group (*P* = 0.0009, Fig. 6E).

### Modulation of PI3K/Akt/mTOR pathway and *Yap-1* through metformin for protection against cyclophosphamide treatment

**Fig. 7:**
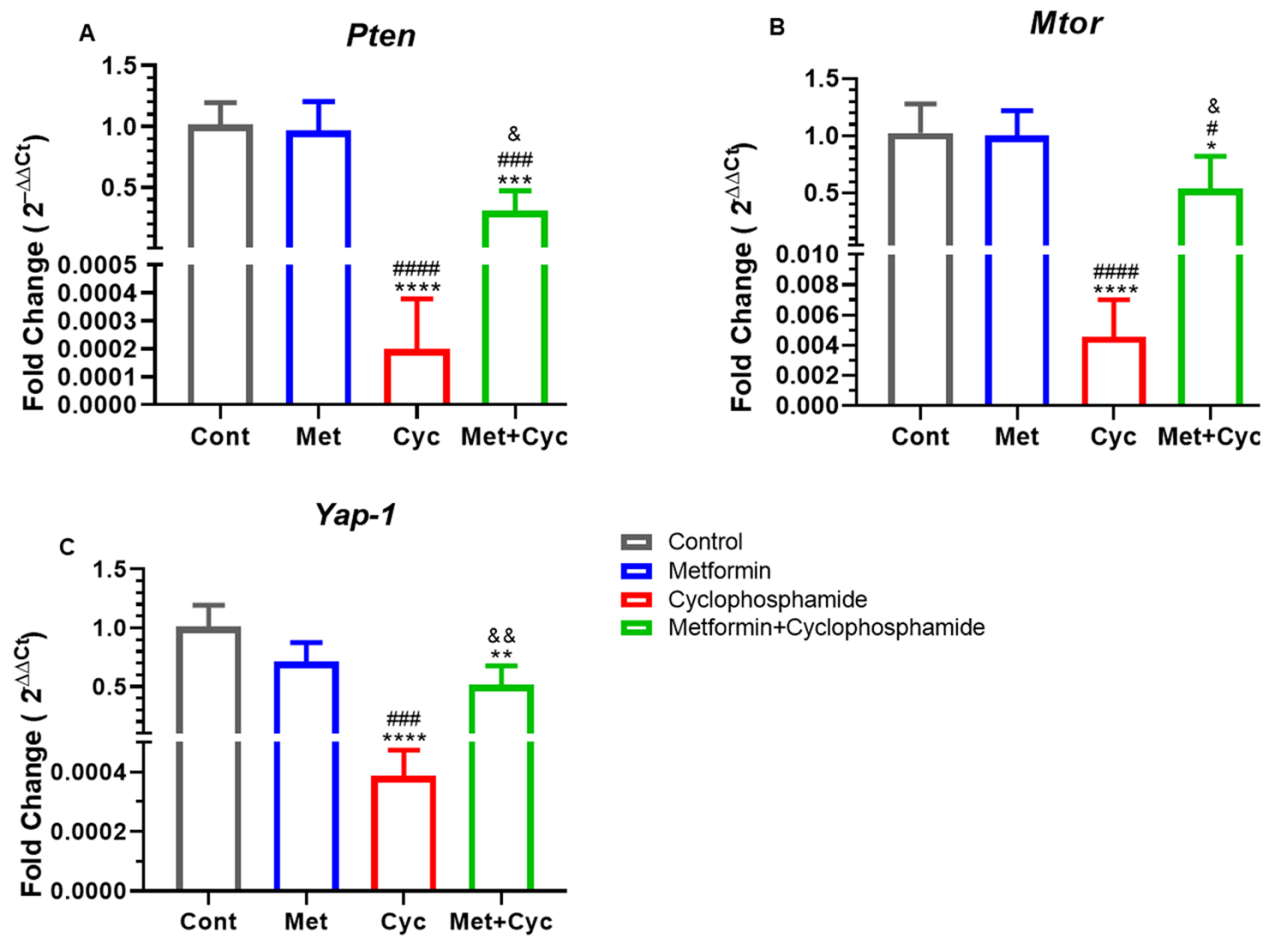
*Pten*, *Mtor* and *Yap-1* genes expression in the four experimental groups. (**A**)*Pten*, (**B**)*Mtor* and (**C**)*Yap-1*. Data are expressed as the mean ± SD. ^*^P < 0.05, ^**^P < 0.01, ^***^P < 0.001 and ^****^P < 0.0001, compared with the Cont group; ^#^*P* < 0.05, ^##^*P* < 0.01, ^###^*P* < 0.001 and ^####^*P* < 0.0001, compared with the Met group and ^&^*p* < 0.05 and ^&&^*p* < 0.01 compared with the Cyc group

From the PI3K/Akt/mTOR pathway, two genes, *Pten* and *Mtor*, as well as the *Yap-1* gene from the Hippo pathway, were analyzed; (Fig. 7A-C). The expression of the *Pten* gene in the Cyc group was significantly decreased compared to the Cont and Met groups (*P* < 0.0001), with improvement noted by treatment with metformin (*P* = 0.0459). Additionally, there was a decrease in *Pten* expression in the Met + Cyc group compared to the Cont and Met groups (*P* = 0.0002, *P* = 0.0003, respectively, Fig. 7A). *Mtor* gene expression exhibited a similar trend to *Pten* gene expression; a significant decrease was observed in the Cyc group compared to all three groups, Cont, Met, and Met + Cyc (*P* < 0.0001, *P* < 0.0001, and *P* = 0.0106, respectively). Moreover, a decrease in the *Mtor* gene expression was noted in the Met + Cyc group compared to the Cont and Met groups (*P* = 0.0287 and *P* = 0.0370, respectively, Fig. 7B). On the other hand, treatment with metformin alongside cyclophosphamide increased the expression of the *Yap-1* gene compared to the Cyc group (*P* = 0.0015). However, both the Met + Cyc and Cyc groups showed a decrease in the expression of the *Yap-1* gene compared to the Cont group (*P* = 0.0042 and *P* < 0.0001, respectively, Fig. 7C).

## Discussion

Emerging evidence indicates that the use of cyclophosphamide in women and girls can lead to reduced ovarian reserve as a side effect [7]. Additionally, it has been suggested that compounds possessing antioxidant properties could be beneficial in protecting ovarian function against the adverse effects of chemotherapy [12]. In this study, we present novel findings demonstrating that the administration of metformin shortly before and after the initiation of chemotherapy in prepubertal mice can aid in preserving ovarian reserve. This preservation is achieved through an increase in the number of ovarian primordial follicles, elevation of blood AMH levels, and regulation of gene expression within the PI3K/Akt/mTOR pathway and *Yap-1* gene from the Hippo pathway.

Several studies have documented those mice treated with cyclophosphamide experience weight loss [26–28]. In our investigation, metformin could potentially offset the weight loss induced by cyclophosphamide. Additionally, mice treated with cyclophosphamide exhibited reduced volume in the medulla, cortex, and whole ovary. Our findings align with those of Dehghani et al., who demonstrated that cyclophosphamide notably decreased cortical volume in rats [29]. Another observed side effect of cyclophosphamide administration is a decrease in AMH hormone levels [28, 30–32]. In a study by Huang et al., it was reported that metformin helped maintain AMH levels in adult mice treated with cyclophosphamide, although the difference was not statistically significant [20]. Furthermore, various in vivo studies utilizing adjuvant agents such as quercetin, Zigui-Yichong-Fang, and melatonin as ovarian protectors against cyclophosphamide have shown a significant increase in AMH levels [30–32]. Consistent with these findings, our study demonstrated that metformin significantly improves AMH levels.

Cyclophosphamide has been shown to reduce the number of primary, secondary, and antral follicles [27, 28] and increase the ratio of growing follicles to primordial follicles, which have detrimental effects on the reproductive system [30]. Our study results indicate that cyclophosphamide, by over-activating primordial follicles, diminishes ovarian reserve. Conversely, metformin, functioning as an ovarian protective agent against cyclophosphamide, enhances follicular condition, thereby preserving ovarian reserve. Specifically, metformin prevents the activation of primordial follicles and reduces the ratio of growing follicles to primordial follicles compared to the cyclophosphamide group. Moreover, a decrease in AMH levels may be associated with a reduction in the number of growing follicles. Furthermore, the ovarian condition in the Met group did not significantly differ from that in the Cont group, suggesting that metformin does not adversely affect the ovaries. Consequently, metformin, akin to several other protective agents such as rapamycin, AS101, quercetin, crocetin, and melatonin, appears to mitigate the negative effects of chemotherapy on ovarian reserve [3, 14, 30, 33, 34].

The present study, consistent with prior research, revealed an augmentation in the number of atretic follicles [30, 35], alongside an upregulation of the *P53* gene [36] and *Bax* gene expression and downregulation of the pro-apoptotic gene *Bcl-2* as additional negative effects of cyclophosphamide on the ovary [37]. Furthermore, Barekati et al. demonstrated that administration of cyclophosphamide leads to decreased *Bax* gene expression and increased expression of *Bcl-2*, *Bclxl*, and *Casp3* genes [38]. Studies have indicated that following cyclophosphamide treatment, there is an increase in BAX protein levels and a decrease in BCL-2 levels [32, 37]. The rise in the TUNEL-positive percentage provides further evidence of chemotherapy drugs’ impact on augmenting apoptosis in the ovary [20, 38]. Moreover, other chemotherapy drugs, such as cisplatin, exert a similar effect by increasing follicular atresia and upregulating the *P53* gene in rat ovaries [39]. In their investigation of protective agents, Wang et al. elucidated acupuncture as a method for preserving ovarian reserve in rats treated with cyclophosphamide. They found that acupuncture reduced BAX expression at both the gene and protein levels while increasing BCL-2 expression at both gene and protein levels [37]. Additionally, among other agents that have mitigated the impact of chemotherapy on follicular apoptosis and the expression of *Bax* and *Bcl-2* genes, Chinese herbal medicines such as Danggui Buxue Decoction have shown promising results [40]. In our study, as anticipated, metformin reduced the number of atretic follicles and significantly downregulated the expression of *P53* and *Bax* genes. Although a decrease in *Bcl-2* expression was observed, it did not reach statistical significance.

An in vitro study conducted by Ganesan et al. demonstrated that the expression of the *Rad-51* gene in rat granulosa cells exposed to the active substance of cyclophosphamide (phosphoramide mustard) is dependent on both the duration and dosage of the drug. They observed that at a dose of 6 µM, there was an initial increase in expression within the first 24 h, but this effect diminished after 48 h, returning to levels incomparable to the control group. This led to the conclusion that the activation of repair pathways is transient, and depending on the dosage and time after drug administration, cells may either continue with the repair pathway or undergo apoptosis [41]. In contrast, an in vivo study conducted on rat testes revealed that the expression of the *Rad-51* gene following treatment with busulfan, another chemotherapy drug, is time-dependent. Specifically, the expression of this gene decreased over time [42]. In our study, we observed a decrease in *Rad-51* gene expression after three cyclophosphamide injections. However, daily administration of metformin both before and after the cyclophosphamide injections contributed to the restoration of *Rad-51* gene expression.

Two pivotal pathways involved in the activation of primordial follicles are the PI3K/Akt/mTOR and Hippo pathways [2]. A study investigating the impact of cyclophosphamide on the expression of genes within the PI3K/Akt/mTOR pathway observed a decrease in the expression of *Pi3k* and *Akt* genes, as well as their protein levels. Conversely, acupuncture was found to enhance the expression of these factors compared to the group treated solely with cyclophosphamide [37]. In another study by Su et al., it was demonstrated that treatment with cyclophosphamide led to a reduction in the expression of *Pten* and *Foxo3a* genes. Conversely, administration of the traditional Chinese compound Dingkun Pill resulted in a significant increase in the expression of the *Pten* gene in these mice [43]. On the other hand, a study conducted in 2022 by Al-Shahat et al. analyzed the effects of cisplatin and found a decrease in the expression of *Pi3k*, *Akt*, *Mtor*, and *Pten* genes, coupled with an increase in *Foxo3* gene expression. The administration of melatonin alongside cisplatin effectively regulated the expression of these genes, contributing to the preservation of ovarian reserve [39]. Moreover, Xu et al. demonstrated that cyclophosphamide reduces the expression of the Hippo pathway at the protein level. Specifically, the expression of LATS1, YAP, and MOB1 factors was observed to decrease. However, the use of melatonin helped to modulate these factors [32]. In our study, cyclophosphamide was found to decrease the expression of *Pten*, *Mtor*, and *Yap-1* genes, which are known to be modulated by metformin. The studies discussed highlight the detrimental impact of chemotherapy drugs on critical signaling pathways that play a role in the activation of primordial follicles, specifically the PI3K/Akt/mTOR and Hippo pathways. The findings consistently reveal a decrease in the expression of essential genes, including *Pi3k*, *Akt*, and *Pten*, after treatment with chemotherapy drugs. Nevertheless, several interventions, such as acupuncture [37], and traditional remedies like Dingkun Pill [43], melatonin [39], and metformin, have demonstrated efficacy in mitigating these adverse effects by promoting the expression of these crucial genes. Furthermore, given that these two pathways are crucial for cell growth and proliferation, any imbalance within them significantly affects cellular development. Our study of prepubertal mice ovaries, conducted on non-adult cases which may response to chemotherapy and protective agents differently, revealed valuable insights into the regulatory role of metformin on the PI3K/Akt/mTOR pathway and possibly Hippo pathway, contributing to ovarian preservation. Metformin, an accessible and affordable drug, could be an alternative therapy for mitigating the chemotherapy-induced harm to ovarian reserve and reproductive health in prepubertal patients, requiring clinical trial confirmation. Nevertheless, further investigation is required, particularly concerning the protein expression of these factors at both phosphorylated and non-phosphorylated levels, in addition to the localization of factors such as FOXO3 and YAP-1.

## Conclusion

Together, metformin exhibits the potential to safeguard ovarian function and fertility against chemotherapy-induced ovarian damage in prepubertal mice by modulating PI3K/Akt/mTOR pathway and the key gene of the Hippo pathway, *Yap-1*. By doing so, metformin helps prevent excessive activation of primordial follicles, reduces apoptosis, enhances repair pathways, and regulates blood AMH levels. Consequently, metformin emerges as a promising candidate for ovarian preservation in girls undergoing treatment with cyclophosphamide. Notably, metformin’s minimal side effects and its capacity to alleviate the condition of cyclophosphamide-exposed ovaries further underscore its potential as an effective therapeutic intervention in this context.

## Data Availability

No datasets were generated or analysed during the current study.

## Abbreviations

POI: Premature Ovarian Insufficiency
PI3K: Phosphoinositide-3-kinase
Akt: Protein kinase B
mTOR: mammalian Target of Rapamycin
FOXO3: Forkhead Box O3
YAP: Yes-Associated Protein
TAZ: Transcriptional coactivator with PDZ-binding
AMPK: AMP-activated protein kinase
AMH: Anti-Müllerian Hormone
ROS: Reactive Oxygen Species
BMI: Body Mass Index
